# Research protocol: EB-GIS4HEALTH UK – foundation evidence base and ontology-based framework of modular, reusable models for UK/NHS health and healthcare GIS applications

**DOI:** 10.1186/1476-072X-4-2

**Published:** 2005-01-13

**Authors:** Maged N Kamel Boulos

**Affiliations:** 1 School for Health, University of Bath, Claverton Down, Bath BA2 7AY, UK

## Abstract

EB-GIS4HEALTH UK aims at building a UK-oriented foundation evidence base and modular conceptual models for GIS applications and programmes in health and healthcare to improve the currently poor GIS state of affairs within the NHS; help the NHS understand and harness the importance of spatial information in the health sector in order to better respond to national health plans, priorities, and requirements; and also foster the much-needed NHS-academia GIS collaboration. The project will focus on diabetes and dental care, which together account for about 11% of the annual NHS budget, and are thus important topics where GIS can help optimising resource utilisation and outcomes. Virtual e-focus groups will ensure all UK/NHS health GIS stakeholders are represented. The models will be built using Protégé ontology editor  based on the best evidence pooled in the project's evidence base (from critical literature reviews and e-focus groups). We will disseminate our evidence base, GIS models, and documentation through the project's Web server. The models will be human-readable in different ways to inform NHS GIS implementers, and it will be possible to also use them to generate the necessary template databases (and even to develop "intelligent" health GIS solutions using software agents) for running the modelled applications. Our products and experience in this project will be transferable to address other national health topics based on the same principles. Our ultimate goal is to provide the NHS with practical, vendor-neutral, modular workflow models, and ready-to-use, evidence-based frameworks for developing successful GIS business plans and implementing GIS to address various health issues. NHS organisations adopting such frameworks will achieve a common understanding of spatial data and processes, which will enable them to efficiently and effectively share, compare, and integrate their data silos and results for more informed planning and better outcomes.

## 

"*Geocoding (street address matching or assignment of latitude and longitude) will be the basis for data linkage and analysis in the 21st century. The versatility of GIS supports the exploration of spatial relationships, patterns, and trends that otherwise would go unnoticed*." – US Healthy People 2010 Objectives (item 23-3 – )

## Introduction

### Geography matters – the need for an evidence-based, spatio-temporal approach to public health

Geography plays a major role in understanding the dynamics of health, and the causes and spread of disease [1]. The classic public health triad composed of man, agent/vehicle and environment emphasises the importance of geographic location (environment or space where we live) in health and disease. Interactions within this triad can also change with time.

Today's health planners aim at developing health policy and services that address geographic and social inequalities in health, and therefore should benefit from evidence-based approaches that can be used to investigate spatial aspects of health policy and practice, and evaluate geographic equity (or inequity) in health service provision [2].

### On geo-information and the need for applications to support the decision maker

According to the US Federal Geographic Data Committee (FGDC), geographic location is a key feature of 80–90% of all government data [3]. The same can be also said about government data in other countries, including data generated by the health sector in the UK. This locational or spatial reference is a "main key" in the transformation of data into information, and for linking and integrating many health and other datasets from disparate sources covering same and contiguous locations [4].

Unlike other resources like employees or funds, spatial data do not suffer any wear and tear from repeated use. On the contrary, reusing data increases the possibilities for improving the content quality of data collections and gaining new insights by linking and exploring the relationships between the different datasets that makeup the big picture [4].

This implies the need to develop applications and not just focus on data. An overemphasis on data acquisition, without health sector-linked applications, will not provide any momentum for further development. Visualisation, modelling and analysing activities will be the focus of value-added services in the coming years [4,5]. Methods must be identified and/or developed to process our spatial data assets to produce meaningful, bottom-line conclusions that can support the decision maker rather than mere bunches of facts. According to Openshaw [5], the ideal methods should be safe and usable by people with no higher degrees in statistical or spatial sciences. The methods should also respond to user needs on the ground, be highly automated, explicitly handle spatial data imprecision, and produce self-evident results that can be mapped and communicated to non-experts.

### On GIS and their health and healthcare applications

In 2003, the US National Library of Medicine added the term "Geographic Information Systems" (GIS) to its controlled vocabulary thesaurus known as MeSH (Medical Subject Headings – see ), a step reflecting the importance and growing use of GIS in health and healthcare research and practices.

The US FGDC defines GIS as "computer systems for the input, storage, maintenance, management, retrieval, analysis, synthesis, and output of geographic or location-based information. In common usage by organisations, GIS include hardware, software, and data. GIS also imply the people and procedures involved in GIS operation" (cited in [6]). The inclusion of "procedures" as part of the above definition is essential for GIS applications in a public health context, given the need to link the science and methods of geographic information science, spatio-temporal statistics, medical geography, epidemiology and public health to GIS output to avoid producing invalid or misleading results [6].

GIS offer a very rich toolbox of methods and technologies that goes far beyond the mere production of simple maps (or digital cartography). From a community health perspective, GIS could potentially act as powerful evidence-based practice tools for early problem detection and solving. When properly used, GIS can be used to: inform and educate (professionals and the public); empower decision-making at all levels; help in planning and tweaking clinically and cost-effective actions, in predicting outcomes before making any financial commitments and ascribing priorities in a climate of finite resources; change practices; and continually monitor and analyse performance and changes, as well as sentinel events.

Traditionally, two broad types of GIS applications can be distinguished which also reflect the two traditions in health geography (geography of disease and geography of healthcare systems), namely health outcomes and epidemiology applications, and healthcare delivery applications. There are also studies at the interface (overlap) between epidemiological and healthcare delivery applications, for example in relation to healthcare commissioning and needs assessment [1,7,8].

### On current issues limiting a wide-scale, optimum adoption of GIS in the UK NHS and many other healthcare systems around the world

The use of GIS in the UK National Health Service (NHS) can still be considered as an emerging technology (in this respect the NHS is not anyway better or more advanced than many other healthcare systems in less developed countries). Despite all their potentials, GIS remain very much under-utilised in the UK NHS in mostly low-level, non-strategic tasks, and in a largely fragmented and uncoordinated way. Spatial data and GIS are still not mentioned in any main UK health information strategy or policy document [7,8]. In striking contrast to this, the US National Health Information Infrastructure Strategy document (also known as "Information for Health") refers explicitly to GIS and real-time health and disease monitoring and states that "public health will need to include in its toolkit integrated data systems; high-quality community-level data; tools to identify significant health trends in real-time data streams; and geographic information systems" (see ). GIS are also explicitly included in the National Electronic Disease Surveillance System (NEDSS) specifications and systems architecture of the US Centres for Disease Control and Prevention (CDC – ).

Although multiple novel spatial statistical and GIS methods are potentially available, we still need to unambiguously determine which method(s) and data specifically should be used by practitioners for each specific health condition of interest, and whether the proposed methods are cost-effective and scalable in the context of UK/NHS settings [8].

Moreover, several researchers have highlighted a gap between academic health-related applications of GIS and their everyday use within the NHS. Research undertaken in academia has certainly highlighted the benefits of spatial statistics and GIS approaches in mapping disease and in healthcare planning, but still needs to respond to NHS needs on the ground [7,8]. On the other hand, it is not uncommon for GIS research to include very practical and useful gems, but these often remain confined to the closed circles of researchers and hidden from the larger communities of GIS professionals and users. The best, current evidence derived from GIS research still needs to be embedded (and regularly updated) in everyday practice and all professional training programmes [8].

In a recent major review paper by this author, the reasons behind the under-utilisation of spatial information and GIS in the NHS, as well as the causes of the gap between academic GIS research and the current everyday use of GIS in the NHS were investigated, and remedial recommendations were made [8]. The reader is referred to this paper for further details.

## Overview and significance of the proposed research

### Research question

How could GIS be beneficial in optimal ways in the context of UK/NHS settings and needs? (Or in other words, how to harness the importance of spatial information in the health sector in order to better respond to national health plans, priorities, and requirements, and to optimise NHS resource utilisation and improve health outcomes.)

Any answer(s) or proposed solutions must be based on the best current evidence in the health GIS field.

### The proposed research

This author proposes to build, consolidate and disseminate a comprehensive evidence base for GIS applications in health and healthcare in the context of UK/NHS settings, and an associated set of evidence-based GIS programme and application models (EB-GIS4HEALTH UK). We will learn from national projects running in the US (a leading country in health GIS) and elsewhere, while ensuring that EB-GIS4HEALTH UK's output properly fits the UK health and healthcare agenda.

**Figure 1:**
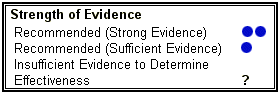
**Key to "Strength of Evidence". **Key to "Strength of Evidence" as displayed within the CDC Community Guide .

A systematic and critical review and consolidation are needed of the evidence for GIS for specific preventable, mitigable and treatable health conditions. A good model is the CDC "Guide to Community Preventive Services" [9]. Topics identified in this guide include alcohol abuse, cancer, diabetes, mental health, motor vehicle occupant injury, oral health, physical activity, sexual behaviour, social environment, tobacco product use, vaccine preventable diseases, and violence. The guide has started building an excellent evidence base, but this does not cover GIS methods and applications (Figure 1).

We propose to address a subset of the topics in the CDC Community Guide (diabetes and dental care – see below) during the three-year period of our project by conducting a focused review of GIS literature on the chosen topics, and then categorising the "nature of the scientific evidence" documenting whether GIS add any value to our understanding and management of the reviewed topics and/or the evidence that it would be feasible and cost-effective for the respective UK NHS and public health programmes tackling the reviewed topics to adopt GIS. Areas requiring further research will be also highlighted. A good example that comes to mind in this context is the 73-page "GIS for cancer" handbook titled "Using Geographic Information Systems Technology in the Collection, Analysis, and Presentation of Cancer Registry Data: A Handbook of Basic Practices" that was published by the North American Association of Central Cancer Registries . However, it should be noted that this particular handbook was conceived with the US healthcare system in mind and is thus more suited to US settings, though still useful as a model to follow.

In reviewing GIS literature for the above mentioned purposes, this author appreciates the fact that the set of definitions and criteria for reviewing evidence as used in the CDC Community Guide is not directly usable for reviewing currently available GIS literature due to the nature of the latter; a modified set of definitions and criteria will first need to be developed. Furthermore, we will organise virtual e-focus groups that bring together UK programme administrators, practitioners and the public to complement the expected gaps and deficiencies in current GIS literature.

The desired GIS information outputs and ways of using them within the NHS will be determined for both diabetes and dental care. Datasets (inputs) and the appropriate processing methods required to reach the desired outputs will be identified driven by the best available evidence (from the literature and e-focus groups), and vendor-neutral GIS programme and application models or ontologies will be created accordingly. Any limitations of these applications and any associated possible data/analysis problems or errors will be also highlighted, along with techniques for recognising and reducing their negative impact on result interpretation and any drawn conclusions.

We will also launch EB-GIS4HEALTH UK Web server to disseminate our results and reach out to the wide NHS audience and the public.

### The project's value and potentials

In a recent study by Higgs *et al*, a substantial proportion of respondents from health authorities (90%) and trusts (74%) stated that a dedicated Web site giving advice on GIS matters for NHS organisations would be helpful in promoting and disseminating good practice examples of GIS use in healthcare [10]. EB-GIS4HEALTH UK Web server will provide this kind of information and much more (for the topics covered during the duration of this project). In this way, EB-GIS4HEALTH UK will be also (partially) addressing the problems of "insufficient guidance" and "lack of awareness of the value of GIS to the NHS" that have been identified in different studies and reviews among the main factors hindering the wider use of GIS within the NHS [7,8,10].

EB-GIS4HEALTH UK will establish links between real-world NHS practice and the growing body of health and healthcare GIS research produced by the academia and research communities to ensure quality GIS practice and innovative applications are developed and implemented in the UK health service.

By putting strong emphasis on real-world, practical GIS scenarios in a UK context, and by being based on the current best evidence, EB-GIS4HEALTH UK will also provide a much needed contribution to future national GIS training and raising awareness campaigns. Such evidence-based training and raising awareness activities have been strongly recommended by different researchers to improve the current poor GIS state of affairs within the NHS [8].

**Figure 2:**
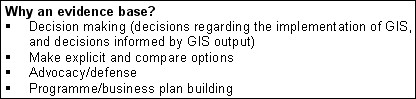
**Why build an evidence base? **Why an evidence base is needed (Modified from ).

Raising awareness activities are also vital given the need to build business cases for the development of GIS within NHS organisations and to show the capabilities and "business benefits" of GIS to directors [10] (Figure 2). EB-GIS4HEALTH UK will act as a vehicle to reach out to policy and strategy makers in the UK health sector and will provide part of the evidence and "proof of concept and benefits" required to gain their support and long-term funding for realising the wider and ultimate vision of a national spatial data and information infrastructure and associated multivariate, GIS-enabled health surveillance services to run alongside and become coupled to the nation-wide integrated electronic health and social care records [8].

EB-GIS4HEALTH UK will provide semantically and procedurally consistent health and healthcare GIS application frameworks. This will help individual NHS organisations adopting such frameworks achieve a common understanding of data and processes (shared semantics), which in turn will enable them to efficiently and effectively share, compare, and integrate their data silos and results at local, regional and national levels for more informed planning, national comparisons, joined-up working, and better outcomes.

### The foundation for a wider vision

EB-GIS4HEALTH UK also forms the basis of our vision of a next-generation intelligent tools specifically designed for the UK public health and NHS, and seamlessly weaved into everyday workflows and decision-making processes to enable users to focus and spend the larger part of their work time on what they want to achieve rather than on learning and overcoming the limitations of tools they are supposed to use to achieve their goals. These tools are part of our wider vision of a "national spatial data and information infrastructure and associated multivariate, GIS-enabled health surveillance services processing real-time or near-real-time data streams rather than just retrospective data" mentioned above and in [8].

Such tools must be able to convey reasonable conclusions (rather than just bunches of facts on thematic, coloured maps). The ideal tools also need to be fault-tolerant and capable of analysing and presenting assembled data in ways that facilitate only appropriate interpretations of integrated data. This can be achieved by using some form of user friendly, "intelligent", goal-oriented health GIS wizards based on robust statistical and epidemiological methods, so that only valid results and maps are produced, even when users attempt to select inappropriate settings for a particular analysis. The tools are also best designed and built to work in modular and nested fashions, so that they may be reused, linked and combined in different ways as needed to serve different scenarios and compound situations with little or no modifications (of the tools) [8].

This vision definitely calls for a sound evidence base and comprehensive conceptual blueprints (the proposed EB-GIS4HEALTH UK evidence-based models) to drive the envisaged tools and wizards.

### Why diabetes and dental care

An incremental approach has been widely recommended in the literature for programmes with a national vision like EB-GIS4HEALTH UK. Rather than addressing all the spectrum of health and healthcare topics that are amenable to GIS processing in one project, we selected only two health conditions to start with. Diabetes and dental care were chosen because of their importance and the huge burden they place on the NHS, and the facts that they affect various age and socio-economic groups and involve a wide spectrum of preventive and curative interventions, which make it possible to apply the GIS models we are proposing to develop for these two topics to many other health and healthcare topics with little modification.

Diabetes has a significant impact on the UK health and social services. Around five per cent of total NHS resources and up to ten per cent of hospital in-patient resources are used for the care of people with diabetes [11]. It has been estimated that diabetes accounts for some nine per cent (approximately £5.2 billion in 2000) of the annual NHS budget [12] (similar figures could be expected in many other countries). Moreover, in the UK, significant inequalities exist in the risk of developing diabetes, in access to health services and the quality of those services, and in health outcomes, particularly with regard to Type 2 diabetics. Reducing health inequalities is a core strand of The NHS Plan and, as the diabetes NSF (National Service Framework) is implemented, particular regard will need to be given to identifying the need for services, including unmet need; planning and delivering services on the basis of need, including reaching those who may not currently be accessing services or are accessing them late; ensuring the active involvement of users in service development; ensuring services are appropriate to individuals' needs, such as ethnicity, language, culture, religion, gender, disability, age and location; performance monitoring; and measuring and monitoring the health inequalities gap to ensure it is narrowing [11]. In all these areas, GIS can assist in analysing the socio-demographic makeup of service areas, and in planning and monitoring interventions and programmes to address these inequalities in the most efficient and effective ways, making sure NHS funds are properly allocated to areas most in need [13,14].

Regarding dental care, the cost of General Dental Services to the government in the year to March 2002 was £1.12 billion (about two per cent of the annual NHS budget of £65 billion in 2002) [15]. Under the recent Health and Social Care (Community Health and Standards) Act 2003, from April 2005 commissioning and contracting for NHS dentistry will devolve from the Department of Health to PCTs (Primary Care Trusts) [16]. Moreover, in September 2003, it was announced that £65.2 million will be made available to improve dental care for NHS patients (see ). Of this latter sum, £35 million will be allocated to enable PCTs to improve access, choice and quality for patients, and £30 million will be directed to information technology to integrate dentistry within the national information technology programme. (The latter has yet to recognise the many potentials of spatial information and GIS for the NHS.)

Profiling service areas and needs assessment, dental healthcare commissioning, and improving/ensuring equitable access to dental services are all areas where GIS can make a positive difference. GIS have significant potential in examining spatial patterns in dental health, and in analysing patterns of registration and utilisation of dental services for different sectors of the community. GIS can reveal gaps in dental health provision, and thus help target resources and programmes to particular areas of needs. GIS can be also used to analyse the composition and spatial distribution of the dental workforce, and to inform the development and monitor the execution of programmes for attracting dental care professionals to work in under-served areas [2,17,18].

### The project's beneficiaries

These include: (1) the NHS and public health services in England (and the UK) – EB-GIS4HEALTH UK aims at adding the missing spatial information dimension to NHS organisations, and informing the development of successful GIS business plans for the health conditions under consideration; (2) the recently established NHSU, the corporate university for the NHS with Special Health Authority status – , could also benefit from EB-GIS4HEALTH UK as a foundation resource to provide evidence-based training programmes to NHS staff in "GIS for health and healthcare" and foster the much-needed NHS-academia collaboration. NHSU currently does not have programmes covering this important area. As an aside, one might add that when the Director of the Medical Informatics programme that the US CDC runs for its new staff conducted a poll of 40 students on the area they most wanted more information about, top of their list was GIS (Gerard Rushton, Department of Geography, University of Iowa, personal communication – December 2003); and (3) the UK citizenry and communities who will be empowered to become more active partners in their healthcare, and will also benefit on the long run from improved health services and outcomes as a result of the introduction of well-founded spatial information management within the NHS through EB-GIS4HEALTH UK and other synergistic/follow-on projects in the future.

## Aim, objectives and methods

### Aim

EB-GIS4HEALTH UK aims at building a foundation evidence base and conceptual models for GIS applications in health and healthcare in the context of UK/NHS settings to (i) improve the currently poor GIS state of affairs within the NHS; (ii) help the NHS understand and harness the importance of spatial information in the health sector in order to better respond to national health plans, priorities, requirements, and NSFs (National Service Frameworks); and also (iii) foster the much-needed NHS-academia GIS collaboration.

### Objectives

1. Organise e-focus groups of representatives of all stakeholders of UK/NHS health GIS to inform the development of all EB-GIS4HEALTH UK products.

2. Review the literature and current UK Public Health/NHS data flows and practices of relevance, and build an evidence base of GIS applications in diabetes and dental care (the two topics chosen for this project) in the context of UK/NHS settings.

3. Build modular GIS programme and application models for diabetes and dental care tailored to UK/NHS settings (driven by the evidence identified through objectives 1 and 2).

4. Run a Web server to disseminate the project's evidence base and resultant GIS programme and application models for diabetes and dental care to the wide NHS audience and the public.

5. Conduct a small-scale formative evaluation of EB-GIS4HEALTH UK server use and potential impact on NHS resource utilisation and improving health outcomes.

## Methods

### I. Process: gathering the evidence; product: building EB-GIS4HEALTH UK evidence base for diabetes and dental care

#### Gaining insight about UK/NHS settings and formulating UK oriented questions

We will gain insight about current Public Health/NHS (in England) data assets, flows and processes of relevance to EB-GIS4HEALTH UK through our NHS collaborators, and by identifying key contacts and organising virtual e-focus groups (see below). This insight will help us come up with a set of "localised" (UK oriented) questions that we will have to answer through our literature review and further e-focus group rounds.

#### Developing a search strategy for locating the evidence and criteria for reviewing it

We will formulate a search strategy to locate potential resources for our evidence base. This will also cover the so-called grey literature. Our search strategy will span MEDLINE/PubMed and other journal resources not listed in PubMed, e.g., Cartography and Geographic Information Science , Transactions in GIS , International Journal of Geographical Information Science , CDC Public Health GIS News and Information , ESRI's HealthyGIS and ArcUser Online ( and ), etc. We will also hand search a range of textbooks on GIS applications in health and healthcare.

We will also review the extensive online documentation of flagship UK and foreign projects and initiatives of relevance to our proposed research. These include, among others, the UK Department of Health NSFs ; the US CDC National Public Health Performance Standards Programme (NPHPSP – ); the US Primary Care Service Area Project (PCSA – ) and the related Dartmouth Atlas project ; the "Mapping A Shared Vision of Hope: The American Indian/Alaska Native (AI/AN) GIS Diabetes Atlas", which was developed to assist in the analysis, design, and evaluation of AI/AN diabetes intervention and prevention programmes (Shirley Baros, Earth Data Analysis Centre, University of New Mexico, personal e-mail communication – January 2004); Georgia Medical Care Foundation Diabetes Quality Indicators project ; and CDC's data systems for oral health .

We will review studies that used GIS or spatial methods in diabetes and dental care (e.g., [2,13,14,17-19]), and studies describing GIS or spatial methods that can be used in diabetes and dental care. The soundness and robustness of all reviewed literature and methods will be assessed and quality of the evidence determined. The conventional set of definitions and criteria for reviewing the evidence in medical literature by distinguishing between case-control, prospective cohort studies, and so forth is not directly usable for reviewing health GIS literature. Most GIS literature is in the form of application/methodology reports and expert opinions/reviews; a modified set of criteria needs to be agreed upon that recognises the different nature of GIS literature and its technical aspects. One thing to look at is whether a given method/approach is successfully replicated in more than one study. This sort of "consensus of opinions" could be an indicator of good evidence quality. Another issue to cover in our reviews is whether a given piece of evidence is suitable to UK/NHS settings and datasets.

#### The virtual e-focus groups

We will organise virtual e-focus groups of UK public health and GIS/informatics programme administrators (including our NHS collaborators), practitioners and representatives of the public to (1) inform the project about NHS settings; (2) define and refine the key questions that decision makers would want to be able to answer with GIS for diabetes and dental care, and think explicitly about what data and methods should be used to answer those questions; (3) discuss the reviewed literature and guide us to any relevant literature we may have missed for our evidence base; (4) complement our review of the evidence by the group members' own experiences and consensus of opinions, especially in areas where the literature is lacking; and (5) provide iterative feedback on EB-GIS4HEALTH UK models, as these are developed and refined. The virtual e-focus groups will ensure that representatives of all stakeholders of UK/NHS health GIS are involved in formulating EB-GIS4HEALTH UK products, a very important ingredient of success [8,20].

The e-focus groups will take place online using threaded discussion technology on EB-GIS4HEALTH UK Web server. Simple audio-conferencing over the Internet, e-mail/e-mail list, and/or telephone may be also used if necessary. The project's team will moderate/facilitate and guide/coordinate the discussions by adopting a kind of Delphi process and/or "cluster analysis" approach (where group members list their ideas, opinions and own experiences, then these are sorted and compiled into "themes" by the moderators and presented back to the group, and further rounds of knowledge distillation and aggregation are carried out as appropriate).

#### The evidence base/metadatabase

A metadatabase of reviewed resources classified according to topics and applications will be created and populated. This will form EB-GIS4HEALTH UK searchable online evidence base (see below). The design of resource records in this metadatabase will be guided by the spirit of the matrix method for conducting and organising literature reviews [21]. A classification system will be developed to easily sort and categorise the reviewed evidence. This could be an adapted version of Hu *et al*'s set of categories they have recently used in a small-scale GIS literature review, with the following top classes/sub-classes among others: data collection method(s) (e.g., field survey, disease surveillance system input, etc.); spatial data analysis method(s) (e.g., visualisation, exploratory, spatio-temporal modelling, etc.); study scale (e.g., country/state, city/town, suburb/district, etc.); resolution/geographic unit of analysis (areas, points, others); study purpose (e.g., identify disease risk factors, disease prediction, resource allocation, etc.); GIS software used; bias of study design/limitations; etc. (Wenbiao Hu and colleagues, Queensland University of Technology, Australia, unpublished report – February 2004). The metadatabase will include fields to record the strength of evidence and bottom-line summaries of the reviewed articles. EB-GIS4HEALTH UK models and their documentation will have cross-links to the underpinning evidence in the online metadatabase.

Suitable input from the virtual e-focus groups will be also included in the evidence base. Resources and findings of insufficient evidence quality will still be documented in the evidence base (with a poor evidence quality rating attached to them), but will not be used in developing EB-GIS4HEALTH UK models.

### II. Process: translate evidence of acceptable strength into recommendations/modular GIS models; product: modular, conceptual GIS programme and application workflow models for diabetes and dental care

#### Developing a general conceptual framework

We will develop a general conceptual framework for EB-GIS4HEALTH UK models as outlined below, guided by Briggs' indicators methodology for environmental health hazard mapping [22,23], the US National Association of County and City Health Officials (NACCHO) core and extended health indicators, which form part of their Community Health Status Assessment (CHSA) Toolbox [24], and the Health Data Model (HDM), a project at the University of California at Santa Barbara (UCSB) to develop a US-oriented data model for health using proprietary ESRI software . EB-GIS4HEALTH UK will take the concepts and methodologies of these projects one step further by developing evidence-based, vendor-neutral modular GIS programme and application models rather than mere data models or vendor-specific solutions.

#### The project's two types of models

**Figure 3:**
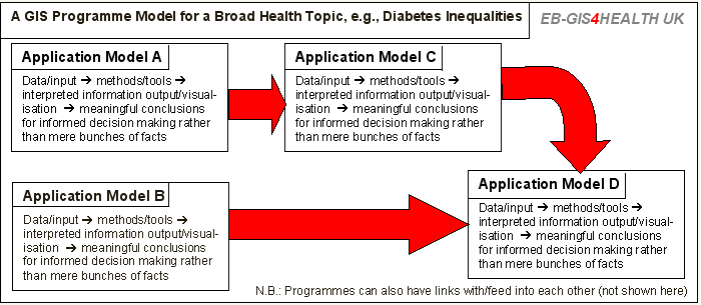
**The project's two types of models. **EB-GIS4HEALTH UK features two interrelated types of models: (1) application models; and (2) programme models, each comprising at least two linked application models. Programme models can also have links with/feed into each other (not shown in this figure).

EB-GIS4HEALTH UK will feature two interrelated types of models: application models and programme models (Figure 3).

An application model comprises (1) data and/or input from other models to be processed; (2) processing methods and tools (methods of geographic information science, spatio-temporal biostatistics, medical geography, epidemiology, health services and public health practice); (3) desired information outputs and output visualisation methods; as well as (4) all valid interpretations/inferences that can be made from the model's output and ways of using them within the NHS. The best current evidence (from the literature and virtual e-focus groups) will be used to formulate all EB-GIS4HEALTH UK models, and will form an integral part of their documentation.

##### Application types

For any single broad health/healthcare topic covered in EB-GIS4HEALTH UK (related to diabetes and dental care), modelled applications can include all or some of the following (among other possibilities): (1) monitoring and measuring NHS performance (many aspects could be measured and analysed such as improvements in the health of the general population, accessibility and utilisation of services, and outcomes of NHS care); (2) surveillance/monitoring/early detection of health and disease patterns and trends in populations; (3) profiling target populations and health service catchment areas; (4) selection of appropriate target groups for public health interventions, optimising these interventions to match target group profiles, and measuring intervention success; and (5) needs assessment and optimising healthcare system planning and responses, including new healthcare facility siting and improving hospital bed availability.

EB-GIS4HEALTH UK models will take into account the influence of non-medical determinants (e.g., income, occupation, and environment) on population health status, qualitatively relate these determinants to health outcomes, and consider their implications on healthcare service planning and delivery [8,20]. For each application model, an ontology (conceptual data and process/workflow model) will be built to capture the properties of, and relationships between the different datasets involved in the application in question, as well as the various data elements (and their relationships) within individual datasets. The ontology will also capture the interactions of application data with all involved processing methods. This will help make explicit all application data requirements and processes, and facilitate data and application integration.

A programme model is also an ontology that addresses a single broad health/healthcare topic, and comprises at least two application models linked together and interacting with each other in predetermined and purposeful ways towards a broader goal than that covered by a single application model. Application models may have uses in more than one programme (either unchanged or with slight modifications to suit different programmes, e.g., different input datasets).

#### The project's ontology editor

**Figure 4:**
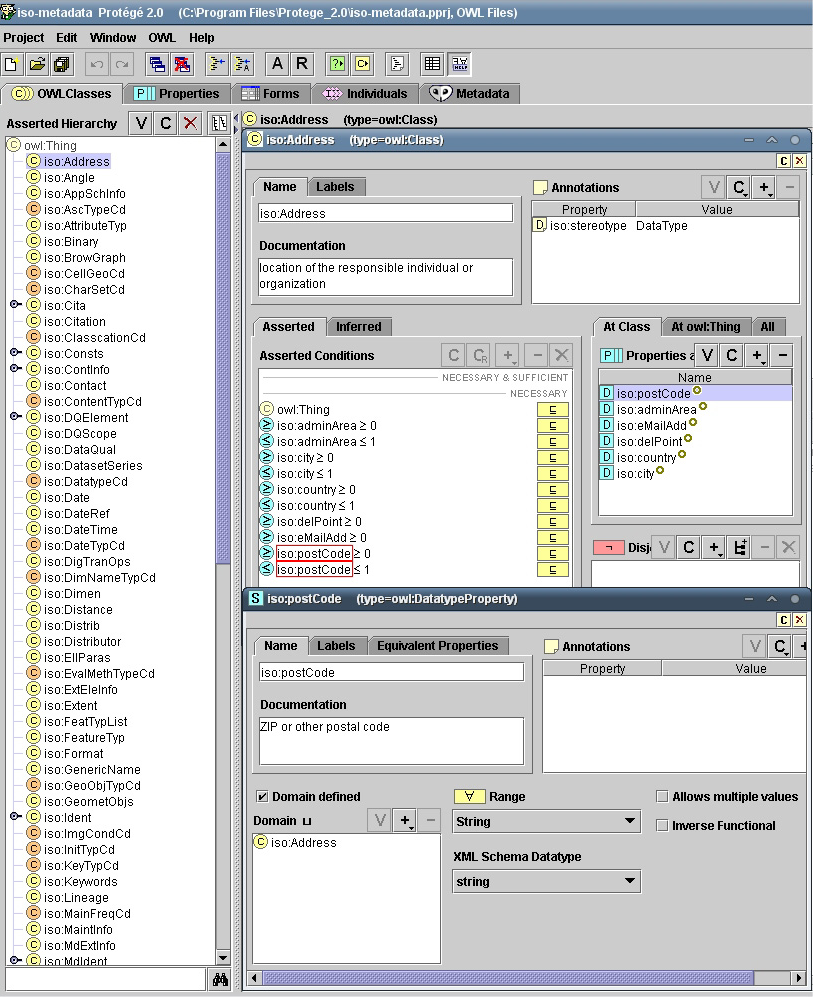
**Protégé screenshot of the geographic information metadata (ISO 19115) ontology. **Protégé screenshot of the geographic information metadata (ISO 19115) ontology developed at Drexel University, US, and available for downloading from . The ontology is distributed in OWL Web Ontology Language format, which is supported by Protégé. OWL is now also an official World Wide Web Consortium (W3C) Recommendation (see ).

EB-GIS4HEALTH UK ontologies (the models) will be built using Protégé , a free ontology modelling tool from Stanford Medical Informatics with which this author has long experience [25,26]. Protégé features an extensive library of free plugins and applications that can extend the tool's basic functionality in many useful ways , including a Java-based Web application for sharing Protégé ontologies over the Web . Protégé is not alien to geographic information science; last year (2003) for example, a geographic information metadata (ISO 19115) ontology was developed at Drexel University, US, using Protégé ( – Figure 4).

#### Anatomy of a model

##### Metadata

A model's ontology/documentation will specify the model's version/date and release history, the programme(s) (for application models)/topic(s)/sub-topic(s) to which the model relates, the model's rationale and role, any alternative or related models/model sets (programmes), links to the underpinning evidence (from the literature/the project's evidence base), and a listing of all agencies/NHS bodies involved in the model's process(es) (and their exact roles).

##### Inputs

Data involved in the modelled applications will be identified and assessed regarding availability, quality, characteristics, and constraints in terms of the model in question. Whenever this is possible, EB-GIS4HEALTH UK models will strive to refer to and model readily available data already collected for other purposes, since implementing new data collection processes can have prohibitive costs, and healthcare workers have repeatedly demonstrated poor compliance with additional data collection and administrative tasks [27].

For data that are not available but are "required" we will investigate and suggest the most efficient and effective way(s) for collecting and maintaining such datasets. During the course of the project, we will be identifying and communicating with the custodians of various datasets in the UK health sector and related sectors, as appropriate, to check the availability of required datasets to the UK NHS, note any constraints related to their release and use (e.g., privacy issues), and flag any need to review policies related to the release of such data. The results of all these data-related investigations will be included in the documentation of EB-GIS4HEALTH UK models.

##### Processing/methods

The spatial and related methods involved in processing the model's input to produce the desired outputs will be described in detail in the model. A reusable toolbox of "generic" methods will be identified to make this process easier for all the models we develop, e.g., "pattern spotters and testers" and "relationship seekers and provers" [5]. All methods used in EB-GIS4HEALTH UK models will be based on documented evidence of acceptable strength, and will be used in ways that allow *only *valid analyses and visualisations of data.

##### Outputs and their interpretation

The model's information output(s), any units of measurements used in presenting outputs, and any specified output visualisation methods will be also described in detail.

The area across which the model can be used (scale of application or aggregation level) will be determined. Finally, the ways in which the model's output may be interpreted (in relation to the topic(s)/sub-topic(s) it covers) and linked to other EB-GIS4HEALTH UK models/programmes will be described. This includes determining what inferences can be made from apparent trends or patterns in the model's output and how such inferences can be used within the NHS, and any constraints on the interpretation of this output, due for example to data limitations or complexities in the relationships implied by the model.

Data and methodological problems and limitations are not uncommon and a wide range of them has been well documented in the literature and must be anticipated and cared for [8]. Techniques for recognising and reducing their negative impact on conclusions drawn from spatial analysis will be investigated and also incorporated into the models.

For a programme model, the component application models will be listed and their relationships and interactions within the programme also documented (Figure 3).

#### What's next

EB-GIS4HEALTH UK knowledge-based models (ontologies) in Protégé will not only be human-readable and visualisable in a variety of ways (to inform NHS GIS service developers and implementers), but it will be also possible to use the models to generate the necessary template databases for running the modelled applications. Furthermore, the resulting ontologies could form the basis for the development of goal-oriented, user friendly and fault tolerant health GIS wizards and solutions in the future using software agents technology [28,29], since we are also representing workflows and methods in our ontologies and not just data.

The goal for EB-GIS4HEALTH UK application and programme models is to provide practical, vendor-neutral modular workflow models and ready-to-use, evidence-based frameworks for implementing GIS to address various health and healthcare topics within the NHS. NHS organisations adopting such frameworks will be able achieve a common understanding of data and processes (shared semantics), which in turn will enable them to efficiently and effectively share, compare, and integrate their data silos and results at all levels.

### III. Process: disseminate the project's evidence base and resultant GIS models for diabetes and dental care to the wide NHS audience and the public; product: EB-GIS4HEALTH UK Web server

We will launch EB-GIS4HEALTH UK public Web server (with it's own domain name) to disseminate our evidence base, GIS models and associated documentation/metadata, as well as other project documentation and recommendations. The server will also host the virtual e-focus groups. The server will run one of the many free content management and discussion board platforms available today to manage and make searchable all of the project's online components.

### IV. Process: formative evaluation of EB-GIS4HEALTH UK online service; product: evaluation report

We will advertise EB-GIS4HEALTH UK online service among target groups (NHS and academia) and the general public, and conduct a small-scale formative evaluation of its use and user-perceived utility of the project using an online user questionnaire, in addition to analysis of EB-GIS4HEALTH UK server transaction logs. This formative evaluation study will be carried during the third year of the project (after the online publication of the project's completed evidence base and associated GIS models for diabetes and dental care) with the goal to inform and guide any further development of EB-GIS4HEALTH UK or similar projects. The study will evaluate the potential impacts of EB-GIS4HEALTH UK on NHS resource utilisation and improving health outcomes, among other things, by surveying participants opinions. Evaluation results will be also available from EB-GIS4HEALTH UK public Web server.

## Methodological and ethical issues

Research into ontology-driven geographic information systems (ODGIS) is very rapidly growing due to the many potential and unique advantages that ODGIS promise [30-36]. We anticipate that the ontological representation of health-related GIS/spatial methods will be one of the most challenging, but also most rewarding parts of our project (the description of data and metadata within EB-GIS4HEALTH UK models will be much easier and more straightforward by comparison). A useful discussion of how GIS methods could be described is presented in [36].

This author also advises the use of Tomlinson's methodology to plan for the successful deployment of GIS within the NHS, but only at a later stage [8,37,38]. Tomlinson's methodology seems a bit "lacking" when it comes to the health sector and that's where EB-GIS4HEALTH UK can come to help by providing a sound foundation upon which the methodology can be successfully applied at a later stage. For example, the "functions" (to use Tomlinson's terminology) and software used for health GIS analyses are a superset of those used in other sectors (e.g., functions provided by tools like  and ). We conduct many complex analyses in health and healthcare to answer much more complicated questions (compared to other sectors), and there are many problematic issues surrounding our analyses like, for example, data confidentiality and Jacquez's famous "gee whiz" effect [8]. But more importantly, NHS users are currently not fully aware of all useful spatio-temporal analysis possibilities available to them (the academia/research-real-world practice/NHS split mentioned earlier), and as such will need a foundation project like EB-GIS4HEALTH UK to help them identify these many possibilities and uses (or "information products" to again use Tomlinson's terminology) that go far beyond the mere production of simple shaded maps to further empower their decision making processes. EB-GIS4HEALTH UK does not raise any ethical issues (in some of the applications to be modelled, the requirement for personal identifiable information will be referred to and modelled, but no actual real data will be used in the models).

## Public engagement in science

Besides being one of the main project beneficiaries, the UK citizenry (and indeed anyone connected to the World Wide Web) will have full access to EB-GIS4HEALTH UK public reports and fully documented products on the project's public Web server. Representatives of the public will be also involved in the project's e-focus groups (see above). Lay audience short summaries of the project's progress and expected benefits in a jargon-free language will be also published on EB-GIS4HEALTH UK public Web server (see Table 1 for an example). Another valid possibility for EB-GIS4HEALTH UK would be to offer a public lecture/day on "the importance of location in health and healthcare, the project's nature and its value". The lecture could perhaps follow the successful model of GIS days .

**Table 1:** EB-GIS4HEALTH UK example English, jargon-free summary for the lay (educated) audience

***EB-GIS4HEALTH UK example English, jargon-free summary for the lay audience*** Factors affecting health vary with location and over time. Geographic Information Systems (GIS) can help us better understand the geography and interactions of health-related events, exposures, and public health/healthcare resources, and also use this understanding to develop optimised prevention and intervention strategies and programmes. A wide range of GIS applications in health and healthcare have been described in the literature that could benefit public health decision makers, practitioners and the public. These GIS applications range from assisting in the early detection of a bioterrorist attack, to understanding and acting on the complex relationships between the environment, socio-economic factors and health, to healthcare needs assessment and the optimum siting of an appropriate new healthcare facility in a given community, and even route optimisation for ambulance vehicles and healthcare professionals doing home visits. However, despite all their potentials, GIS remain very much under-utilised in the NHS in mostly non-strategic tasks, and in a largely fragmented and uncoordinated way. Geographic data and GIS are still not mentioned in any main UK health information strategy or policy document, in striking contrast to the corresponding US strategy documents and specifications, which explicitly mention GIS. EB-GIS4HEALTH UK aims at helping the NHS understand and harness the importance of spatial information in the health sector in order to better respond to national health plans, priorities, and requirements. Virtual e-focus groups that include representatives of the public will inform the development of all EB-GIS4HEALTH UK products. These include a sound evidence base of GIS methods and applications that are relevant to UK practices and settings, and an associated set of evidence-based conceptual models or blueprints for developing successful GIS business plans and implementing GIS to address various health issues within the NHS. The project will focus on diabetes and dental care, which together account for about 11% of the annual NHS budget, and are thus important topics where GIS can help optimising resource utilisation and outcomes. However, products and experience gained in this project will be transferable to address other national health topics based on the same principles. EB-GIS4HEALTH UK ultimate beneficiaries are the UK citizenry and communities who will be empowered to become more active partners in their healthcare, and will also benefit on the long run from improved health services and outcomes, and reduced health inequalities as a result of the introduction of well-founded geographic information management within the NHS through this and other synergistic/follow-on projects.

## Commercial exploitation

Most major GIS vendors and solution providers have been targeting the UK health sector for many years now (e.g.,  and ), but this has never resulted in any coherent national strategic adoption of GIS within the NHS. This is due to the fact that a successful national strategic implementation of GIS in the health sector is dependent upon many other very important ingredients besides the acquisition of hardware and software systems, and core digital geo-datasets [8]. This author believes that these GIS vendors and solution providers will be definitely interested in the output of EB-GIS4HEALTH UK, which should provide them (and the NHS) with a convincing application-oriented framework for tailoring their services to suit the requirements of a wide-scale strategic adoption of GIS by the UK health sector.

## Concluding remarks

Healthcare systems work differently around the world and this has knock-on effect on health GIS. When it comes to UK health GIS, we still need to go back to the design board, and to link/integrate what research has to offer into workflow models and "recipes" that are both usable and useful in everyday practice *in this country (England)*. All national health GIS stakeholders must be involved in this process, including representatives of the general public (the ultimate beneficiaries). EB-GIS4HEALTH UK is just the start towards this goal. An incremental approach has been widely recommended in the literature for programmes with a national vision like EB-GIS4HEALTH UK. The experience gained at the end of this project will hopefully be transferable to further develop the covered topics (diabetes and dental care – in subsequent follow-on projects), and for addressing other national health and healthcare topics not initially covered in this project, based on the same principles.

It must be emphasised that the evidence base and conceptual GIS workflow models we are proposing to build in this project are not *per se *our ultimate goals. The actual purpose of this design/modelling exercise and its ultimate goals are optimising NHS resource utilisation and improving health outcomes by paving the way to the incorporation of the missing spatial information dimension in NHS organisations.

This paper presented an overview of a project we are proposing to carry out here in England, where the author is based. However, the same concepts, principles and approaches described in this paper can be also applied in other countries, especially where financial constraints are not a major issue. The task might not be as easy as replacing "UK" in this proposal with another country name, but the author believes that the paper has provided enough details and set an example to enable "quick starting" similar health GIS foundation projects in other countries.

Our proposal also fits very well into the spirit of Mark Musen's philosophical paper on medical informatics as an academic discipline, in which he argued that "informatics involves the construction of ontologies that define the concepts relevant to different aspects of human experience and the elucidation of problem-solving methods that can solve specific computational tasks" [39].

## Notes

A detailed breakdown of human and other resources required to complete this project with a timeline of the main EB-GIS4HEALTH UK execution tasks distributed over a suggested three-year duration of the project are not provided in this manuscript, but are directly available from the author.

A slightly abridged version of this proposal has been submitted to, and very favourably reviewed by the UK Medical Research Council (MRC) during 2004, and received a final ALPHA-C banding (i.e., "work which is nationally competitive and will make valuable contributions to addressing important scientific and/or policy questions" – ).

As one of the reviewers rightly noted, "the key aspect to this proposal is that it deals with an area that cannot be neatly fitted into a single category. It crosses over into a number of specialisms – health informatics, geographic information science (GIS), health, geography, public health, policy and practice, epidemiology, etc. It is therefore important to bear in mind that if the proposal is judged by criteria generally applicable to only one or two of these specialist areas, it might be found deficient and it would be quite inappropriate to do this".
